# Minimally invasive bleb surgery versus minimally invasive glaucoma surgery: a 12-month retrospective study

**DOI:** 10.1038/s41598-024-61811-y

**Published:** 2024-06-04

**Authors:** Joanna Konopińska, Kinga Gołaszewska, Emil Saeed

**Affiliations:** Department of Ophthalmology, Medical University of Białystok, Białystok, Poland

**Keywords:** Diseases, Health care, Medical research

## Abstract

This study aimed to compare the IOP-lowering effectiveness and safety of standalone Preserflo MicroShunt and iStent 1st generation implantation combined with phacoemulsification in Caucasian patients with a 12-month follow-up period. This retrospective study analyzed the medical histories of patients undergoing antiglaucoma surgery at the Department of Ophthalmology, Medical University of Bialystok, between January 2019 and January 2022. The main outcome measures were success rates (complete: proportion of eyes with IOP < 18 mmHg (criterion A) and < 15 mmHg (criterion B) or 20% reduction in IOP without any glaucoma medication; qualified: proportion of eyes achieving IOPs < 18 mmHg and < 15 mmHg or 20% reduction in IOP from baseline with or without medications), mean reduction (%) in IOP, medication burden, number of complications and additional interventions. In both groups, a significant decrease in IOP and medication burden were observed at 6 and 12 months when compared with baseline. At 12 months, qualified surgical success in criterion A was recorded in 67.4% and 85.7% of patients in the Preserfo and iStent groups, respectively (p = 0.045). Complete surgical success in criterion B at 12 months accounted to 61.4% of patients from Prserflo group and 32.7% patients in iStent group (p = 0.04). Surgical failure at 12 months was documented in 30.2% and 6.3% of patients, respectively (p = 0.003). There was a significant difference between groups in %IOP reduction after 12 months. Greater reduction was observed in Preserflo group, MD = − 8.41 CI_95_ [− 15.88; − 0.95], p = 0.028, (− 33.49% ± 21.59 vs − 25.07% ± 14.15 in iStent group). Both procedures effectively reduced IOP and postoperative use of antiglaucoma medications in glaucoma patients.

## Introduction

In past decades, a major revolution has occurred in the surgical treatment of glaucoma. From conventional glaucoma filtration surgeries, namely trabeculectomy and glaucoma-draining devices, which are often associated with intra- and postoperative complications and lengthy recovery periods^1–4^, safer alternatives such as microinvasive glaucoma surgeries (MIGS) are now available to the patients. The term MIGS was first introduced in 2012 to describe a group of glaucoma surgeries that share five common features, including a high safety profile, minimal tissue trauma, rapid recovery, at least modest efficacy, and a microinvasive (ab-interno) approach^5^. The first implant that fulfilled these criteria was the first-generation iStent (Glaukos Corporation, Laguna Hills, CA, USA), registered for patients with primary open-angle glaucoma (POAG), pseudoexfoliation glaucoma (PXG), and pigmentary glaucoma (PG), authorized in 2012. The iStent is implanted into the trabecular meshwork, which improve the natural aquas outflow^6^. The first-generation iStent is an L-shaped device with a pipe-like opening placed in the anterior chamber via ab-interno approach. While the device is small (1 mm in length, 0.33 mm in width, 0.25 mm in length of the pipe), according to mathematical models, its lumen is large enough to reduce IOP effectively^7^. The iStent inject is different from the first-generation model^8–11^.

In 2014, the American Glaucoma Society and the US Food and Drug Administration (FDA) expanded MIGS definition to all procedures performed via ab interno or ab externo approaches that require minimal to no scleral dissection^6^. This change broaden the classification of MIGS, adding a new category: minimally invasive bleb surgery (MIBS)^7–9^. Preserflo™ MicroShunt (Santen, Osaka, Japan), authorized in Europe in 2019, bypasses the conventional outflow pathway and conduct aqueous humor from the anterior chamber to the subconjunctival and sub-Tenon spaces using a filtering bleb. Similar to the Xen Gel Stent, the construction of the Preserflo™ MicroShunt was derived from the Hagen-Poiseuille equation^12^. The device has 8.5 mm in length and 1.1 mm in width, with external and internal diameters of 0.70 mm and 0.35 mm, respectively. The small dimensions and permanent location within the scleral pocket enable the device to create a high-filtering bleb posterior to the scleral limbus, which is more comfortable for the patient and less prone to scarring than a bleb obtained after trabeculectomy^13^. The implant is made of a synthetic poly(styrene-block-isobutylene-block-styrene) (SIBS) polymer^14,15^, which easily adjusts to the curvature of the eye, reducing the risk of scleral erosion by the implant.

Studies that directly compare MIGS and MIBS are scarce. To the best of our knowledge, only one comparative study has been conducted on trabecular-MIGS (iStent) and filtering procedures (Preserflo Microshunt)^16^. One potential explanation for this is that MIGS and MIBS have different target populations. The MIGS are typically tailored for patients with mild-to-moderate glaucoma (usually under medical control), low risk of progression, and non-compliance; while MIBS are more suitable for moderate-to-severe glaucoma (usually medically uncontrolled) with a substantial risk of progression. However, there are some indications in which both surgeries can be considered, for instance medically uncontrolled patients with mild-to-moderate disease and medically controlled patients with advanced disease^17^.

The present study aimed to compare the hypotensive effectiveness and safety of Preserflo and iStent implantation combined with phacoemulsification in Caucasian patients during a 12-month follow-up period.

## Methods

This study was based on a retrospective analysis of the medical histories of all consecutive patients who qualified for glaucoma surgeries, either iStent implantation combined with phacoemulsification or Preserflo MicroShunt implantation, at the Department of Ophthalmology, Medical University of Bialystok, and operated between January 2019 and January 2022.

The study was conducted in accordance with the principles of the Declaration of Helsinki. The implantation procedure was approved by the Bioethics Committee of the Medical University of Bialystok (APK.002.581.2021). Informed consent was waived by the Bioethics Committee of the Medical University of Bialystok due to the retrospective character of the study. All patient data were anonymized before statistical analysis of the results.

The qualification criterion for the procedure was the inability to achieve the target intraocular pressure (IOP)^16^ in patients with either mild or moderate POAG or PXG, despite the implementation of maximally tolerated pharmacotherapy (up to three antiglaucoma medications). In the case of iStent implantation, glaucoma patients who reached the target IOP also qualified as long as they required scheduled cataract surgery. The contraindications for surgical treatment included angle-closure glaucoma (ACG), inflammatory glaucoma, neovascular glaucoma, traumatic glaucoma, angle recession, filtration angle dysgenesis, and posterior adhesions. Additional exclusion criteria were a history of glaucoma surgery and a follow-up period of < 12 months. The implantation was not preceded by a washout period.

The analysis included patient demographics, such as sex and age, along with preoperative characteristics, such as glaucoma type, baseline IOP, best-corrected visual acuity in Snellen decimal (BCVA), number and type of antiglaucoma medications, mean deviation (MD), and pattern standard deviation (PSD) on the preoperative 30–2° Humphrey visual field (VF) test. Glaucoma severity was classified as mild (MD > − 6 dB), moderate (MD − 6 to − 12 dB), or severe (MD < − 12 dB).

### Surgical technique

Implantation of the iStent was performed simultaneously with phacoemulsification, as described previously^35^, with the cataract procedure followed by implantation of the device to the trabecular meshwork within the inferonasal quadrant.

Implantation of the Preserflo MicroShunt was performed as a single procedure using the technique described in our previous study^13^.

All surgeries were performed under topical anesthesia with additional conjunctival administration of 2% Xylocaine in Preserflo MicroShunt group.

All patients received topical antibiotics postoperatively three times daily for seven days and topical steroids four times daily; the latter was gradually tapered over a 4–5-week period. Antiglaucoma medications were discontinued after the procedure, but when needed, they were prescribed again following EGS rules^16^.

The analysis included data from control visits before the procedure and at 6 and 12 months thereafter. The main outcome measures were: (1) success rates (complete: no need for repeated glaucoma surgery, and IOP ≤ 18 mmHg (criterion A), ≤ 15 mmHg (criterion B) or 20% reduction from baseline^36^, and discontinuing all antiglaucoma medications; qualified: no need for repeated glaucoma surgery, IOP ≤ 18 mmHg (criterion A), IOP ≤ 15 mmHg (criterion B) or 20% reduction from baseline, with discontinuation of all antiglaucoma medications or without; (2) mean reduction (%) in IOP, (3) medication burden, (4) number of complications and additional interventions. Surgical failure was defined as the need for another glaucoma surgery at any time after the primary procedure or an IOP > 18 mmHg during two consecutive control visits (at 6 and 12 months) despite administering antiglaucoma medications.

### Statistical analysis

Sample size estimation: A minimum of 42 eyes were required to detect a 2.5 mmHg between-group difference in the mean decrease in IOP at twelve months after the procedure, with a significance level and 0.80 power of 0.05, assuming a standard deviation for the IOP decrease in both groups at 4.0 mmHg^37^.

Analysis was performed using the R package version 4.1.2. Categorical variables are summarized as numbers and percentages, and numerical variables as arithmetic means, standard deviations, medians, and interquartile ranges. The normal distribution of the study variables was verified using the Shapiro–Wilk test and confirmed based on skewness and kurtosis values. The homogeneity of variances was verified using Levene’s test. Between-group comparisons were based on Pearson’s chi-square test, Student’s t-test for independent groups, Welch’s t-test for independent groups, and the Mann–Whitney U-test. The significance of within-group changes over time was verified using t-tests for dependent groups and Wilcoxon’s test. Statistical significance for all analyses was set at α = 0.05.

## Results

### Baseline characteristics of the study groups

The analysis included two groups of patients: Preserflo group (N = 43) and iStent group (N = 78). Most patients in both groups were women (69.8% and 67.9%, respectively), with no statistically significant between-group differences in sex distribution (p = 0.999). The mean age of patients from the Preserflo and iStent groups was 68.98 ± 10.09 years and 72.48 ± 8.70 years, respectively, with those from the Preserflo group being significantly younger, by 3.5 years on average (MD = − 3.50, CI_95_ [− 7.00; 0.00], p = 0.049). In addition to age, the groups differed significantly in terms of the number of antiglaucoma medications and baseline IOP, which were higher in Preserflo group (MD = 0.00, CI_95_ [0.00; 1.00], p = 0.027 and MD = 2.00, CI_95_ [1.00; 4.00], p = 0.002, respectively, Table 1).

**Table 1:** Baseline characteristics of the study groups.

Variable	Preserflo		iStent		MD (95% CI)	p
	n (%)/Mean ± SD	Median (Q1;Q3)	n (%) / Mean ± SD	Median (Q1;Q3)		
N	43 (100.0)	–	78 (100.0)	–	–	–
Sex, female	30 (69.8)	–	53 (67.9)	–	–	0.999^3^
Age, years	68.98 ± 10.09	70.00 (61.25; 75.00)	72.48 ± 8.70	75.00 (67.00; 79.00)	− 3.50 (− 7.00; 0.00)^1^	0.049^2^
POAG	34 (79.1)	–	63 (80.8)	–	–	> 0.999^3^
PXG	9 (20.9)	–	15 (19.2)	–		
Medication number	2.24 ± 1.32	2.00 (2.00; 3.00)	1.79 ± 1.03	2.00 (1.00; 2.00)	0.00 (0.00; 1.00)	0.027
BCVA	0.59 ± 0.33	0.60 (0.35; 1.00)	0.56 ± 0.56	0.50 (0.34; 0.68)	0.10 (− 0.03; 0.20)	0.281
IOP, mmHg	25.14 ± 6.53	23.00 (21.00; 28.00)	21.94 ± 2.95	21.00 (20.00; 23.00)	2.00 (1.00; 4.00)	0.002

BCVA, best corrected visual acuity in Snellen decimal score; IOP, intraocular pressure.

Note: SD, standard deviation; Q1, lower quartile; Q3, upper quartile; MD—mean^1^ or median difference (Preserflo vs. iStent). Data presented as n (%) for categorical variables and means ± SD and medians (Q1; Q3) for numerical variables. Between-group comparisons were conducted using Student’s t-tests for independent groups^2^ or Mann–Whitney U-tests (for numerical variables) and Pearson’s chi-square tests^3^ (for categorical variables).

### Comparison of parameters recorded at 6 and 12 months in the Preserflo group

The significance of changes over time in the analyzed parameters was analyzed separately within each study group. In the Preserflo group, the number of antiglaucoma medications decreased significantly between baseline and control visits at both 6 and 12 months (MD = − 1.94, CI_95_ [− 2.40; − 1.49], p < 0.001 and MD = − 1.61, CI_95_ [− 2.10; − 1.11], p < 0.001, respectively). No statistically significant difference in BCVA was observed when the values obtained at 6 and 12 months were compared with baseline measurements (p = 0.061). A significant decrease in baseline IOP was observed at 6 and 12 months (MD = − 9.54, CI_95_ [− 11.90; − 7.17], p < 0.001; and MD = − 9.09, CI_95_ [− 11.26; − 6.92], p < 0.001, respectively; Table 2).

**Table 2:** Changes in the study parameters over time within the Preserflo group.

Variable/measurement	n	Mean ± SD	Median (Q1; Q3)	MD (95% CI)	p
Number of antiglaucoma medications					
Baseline	43	2.24 ± 1.32	2.00 (2.00; 3.00)	–	–
6 months	41	0.29 ± 0.72	0.00 (0.00; 0.00)	− 1.94 (− 2.40; − 1.49)	< 0.001
12 months	43	0.56 ± 1.08	0.00 (0.00; 0.50)	− 1.61 (− 2.10; − 1.11)	< 0.001
BCVA					
Baseline	43	0.59 ± 0.33	0.60 (0.35; 1.00)	–	–
6 months	41	0.68 ± 0.33	0.80 (0.40; 1.00)	0.15 (0.05; 0.35)^1^	0.019^2^
12 months	43	0.68 ± 0.34	0.80 (0.40; 1.00)	0.15 (− 1.23; 3.50)^1^	0.061^2^
IOP, mmHg					
Baseline	43	25.14 ± 6.53	23.00 (21.00; 28.00)	–	–
6 months	41	15.63 ± 4.44	14.00 (13.00; 19.00)	− 9.54 (− 11.90; − 7.17)	< 0.001
12 months	43	16.05 ± 4.60	15.00 (13.00; 19.50)	− 9.09 (− 11.26; − 6.92)	< 0.001

BCVA, best corrected visual acuity in Snellen decimal score, IOP, intraocular pressure.

Note: SD, standard deviation; Q1, lower quartile; Q3, upper quartile; MD, mean or median^1^ difference (consecutive measurements vs. baseline). Within-group comparisons were conducted using t-tests, the Wilcoxon test for dependent samples, or Wilcoxon’s tests^2^. The number of observations (n), mean ± SD and median (Q1; Q3) are given for all available observations, MD (95% CI) and p-values are based on the pairs of observations collected at baseline and during a consecutive measurement.

### Comparison of baseline parameters and parameters recorded at 6 and 12 months in the iStent group

In the iStent group, the number of antiglaucoma medications at both 6 and 12 months was significantly lower than at baseline (MD = − 1.42, CI_95_ [− 1.66; − 1.17], p < 0.001 and MD = − 1.29, CI_95_ [− 1.51; − 1.06], p < 0.001, respectively). The mean BCVA at six months was 0.39 higher than that at baseline (MD = 0.39, CI_95_ [0.33, 0.45], p < 0.001). The BCVA at 12 months was also significantly higher than that at baseline (MD = 0.40, CI_95_ [0.34, 0.48], p < 0.001), likely attributable to cataract extraction. Additionally, a significant decrease in IOP was observed at both 6 and 12 months when compared with the baseline value of this parameter (MD = − 6.50, CI_95_ [− 7.00; − 5.50], p < 0.001 and MD = − 5.50, CI_95_ [− 6.50; − 4.50], p < 0.001, respectively; Table 3).

**Table 3:** Changes in the study parameters over time within the iStent group.

Variable/measurement	n	Mean ± SD	Median (Q1;Q3)	MD (95% CI)	p
Number of antiglaucoma medications					
Baseline	78	1.79 ± 1.03	2.00 (1.00; 2.00)	–	–
6 months	78	0.37 ± 0.74	0.00 (0.00; 0.00)	− 1.42 (− 1.66; − 1.17)	< 0.001
12 months	78	0.46 ± 0.84	0.00 (0.00; 1.00)	− 1.29 (− 1.51; − 1.06)	< 0.001
BCVA					
Baseline	78	0.56 ± 0.56	0.50 (0.34; 0.68)	–	–
6 months	78	0.91 ± 0.18	1.00 (0.90; 1.00)	0.39 (0.33; 0.45)^1^	< 0.001^2^
12 months	78	0.91 ± 0.17	1.00 (0.88; 1.00)	0.40 (0.34; 0.48)^1^	< 0.001^2^
IOP, mmHg					
Baseline	78	21.94 ± 2.95	21.00 (20.00; 23.00)	–	–
6 months	78	15.31 ± 2.77	15.00 (13.00; 17.00)	− 6.50 (− 7.00; − 5.50)^1^	< 0.001^2^
12 months	78	16.06 ± 2.62	16.00 (15.00; 17.25)	− 5.50 (− 6.50; − 4.50)^1^	< 0.001^2^

BCVA, best corrected visual acuity in Snellen decimal score; IOP, intraocular pressure.

Note: SD, standard deviation; Q1, lower quartile; Q3, upper quartile; MD, mean or median^1^ difference (consecutive measurements vs. baseline). Within-group comparisons were conducted using t-tests or the Wilcoxon test for dependent samples or Wilcoxon’s tests^2^. The number of observations (n), mean ± SD and median (Q1; Q3) are given for all available observations, MD (95% CI) and p-values are based on the pairs of observations collected at baseline and during a consecutive measurement.

### Between-group comparison of study parameters

Complete surgical success (≤ 18 mmHg) at 6 months was documented in 63.4% of patients in the Preserflo group and 70.8% from the iStent group. Complete surgical success at 12 months was recorded in 60.5% and 66.7% of the patients in the Preserflo and iStent groups, respectively. No statistically significant differences in the complete surgical success rates were found between the study groups at 6 and 12 months (p = 0.564 and p = 0.655, respectively, Table 4).

**Table 4:** Comparison of surgical success/failure rates in the Preserflo and iStent groups.

Variable	Preserflo	iStent	p
Complete surgical success Criterion A			
6 months	27 (63.4)	55 (70.8)	0.564
12 months	26 (60.5)	52 (66.7)	0.655
Qualified surgical success Criterion A			
6 months	31 (73.2)	68 (87.7)	0.101
12 months	29 (67.4)	66 (85.7)	**0.045**
Complete surgical success Criterion B			
6 months	27 (63.4)	33 (42.8)	0.564
12 months	26 (61.4)	25 (32.7)	**0.04**
Qualified surgical success Criterion B			
6 months	26 (61.1)	24 (31.6)	0.101
12 months	22 (53.5)	28 (35.9)	0.110
Surgical failure			
12 months	13 (30.2)	4 (6.3)	**0.003**

Significant values are in [bold].

Note: Data presented as n (%). Between-group comparisons were conducted using Pearson’s chi-square test.

Criterion A: IOP ≤ 18 mmHg, Criterion B: IOP ≤ 15 mmHg.

Qualified surgical success (≤ 18 mmHg) at 6 months was documented in 73.2% of patients in the Preserflo group and 87.7% from the iStent group; the difference was not statistically significant (p = 0.101). At 12 months, qualified surgical success was recorded in 67.4% and 85.7% of patients in the Preserflo and iStent groups, respectively, with a statistically significant difference (p = 0.045).

Complete surgical success (≤ 15 mmHg) at 12 months accounted to 61.4% of patients from Prserflo group and 32.7% patients in iStent group (p = 0.04).

Qualified surgical success (≤ 15 mmHg) was achieved in 53.5% of patients in the Preserflo group and 35.9% in the iStent group at 12 months. However, this difference was not statistically significant (p = 0.110).

Surgical failure at 12 months was documented in 30.2% (n = 13) of patients in the Preserflo group and 6.3% (n = 4) of patients in the iStent group, with a statistically significant difference (p = 0.003), Table 4.

There was a significant difference between groups in %IOP reduction after 12 months. Greater reduction was observed in Preserflo group, MD = -8.41 CI_95_ [− 15.88; − 0.95], p = 0.028 (− 33.49% ± 21.59 vs − 25.07% ± 14.15). Statistical difference between groups was not confirmed for IOP reduction after 6 months, Table 5.

**Table 5:** Comparison of IOP reduction between groups.

Variable	Preserflo		iStent		MD (95% CI)	p
	Mean ± SD	Median (Q1;Q3)	Mean ± SD	Median (Q1;Q3)		
IOP change vs baseline, %						
After 6 months	− 34.75 ± 23.03	− 39.13 (− 50.00; − 22.22)	− 29.35 ± 13.46	− 28.57 (− 36.36; − 22.73)	− 5.40 (− 13.34; 2.54)	0.178
After 12 months	− 33.49 ± 21.59	− 34.78 (− 50.00; − 22.22)	− 25.07 ± 14.15	− 24.40 (− 32.20; − 19.05)	− 8.41 (− 15.88; − 0.95)	**0.028**

BCVA, best corrected visual acuity in Snellen decimal score; IOP, intraocular pressure.

SD, standard deviation; Q1, 1st quartile; Q3, 3rd quartile; MD, mean difference (Preserflo vs iStent). Groups comparison performer with independent t-Welcha test. Significant values are in bold.

Other outcomes measure are shown in Table 6.

**Table 6:** Between-group comparison of the study parameters.

Variable	Preserflo		iStent		MD (95% CI)	p
	Mean ± SD	Median (Q1;Q3)	Mean ± SD	Median (Q1;Q3)		
Medication number 6 M	0.29 ± 0.72	0.00 (0.00; 0.00)	0.37 ± 0.74	0.00 (0.00; 0.00)	0.00 (0.00; 0.00)	0.538
BCVA 6 M	0.68 ± 0.33	0.80 (0.40; 1.00)	0.91 ± 0.18	1.00 (0.90; 1.00)	− 0.20 (− 0.25; 0.00)	< 0.001
IOP 6 M, mmHg	15.63 ± 4.44	14.00 (13.00; 19.00)	15.31 ± 2.77	15.00 (13.00; 17.00)	0.32 (− 1.23; 1.87)^1^	0.679^2^
Medication number 12 M	0.56 ± 1.08	0.00 (0.00; 0.50)	0.46 ± 0.84	0.00 (0.00; 1.00)	0.00 (0.00; 0.00)	0.957
BCVA 12 M	0.68 ± 0.34	0.80 (0.40; 1.00)	0.91 ± 0.17	1.00 (0.88; 1.00)	− 0.20 (− 0.30; 0.00)	< 0.001
IOP 12 M, mmHg	16.05 ± 4.60	15.00 (13.00; 19.50)	16.06 ± 2.62	16.00 (15.00; 17.25)	− 1.00 (− 2.00; 1.00)	0.514

BCVA, best corrected visual acuity in Snellen decimal score; IOP, intraocular pressure; M, month.

Note: SD, standard deviation; Q1, lower quartile; Q3, upper quartile; MD, mean^1^ or median difference (preserflo vs. iStent). Between-group comparisons were conducted using Welch’s t-tests for independent groups^2^ or Mann–Whitney U-tests.

### Safety

The intraoperative and early and late postoperative complication rates in both groups are presented in Table 7.

**Table 7:** Intraoperative complications, early and late postoperative complications stratified according to the study group.

	iStent (%)	Preserflo (%)
Intraoperative complications and early postoperative complications		
Microhyphema (< 1 mm of blood in the anterior chamber)	11%	0
Elevated IOP	4.3%	0
Corneal erosion	0	5%
Hypotony	0	10%
Choroidal detachment	0	2.55%
Keratopathy	0	5%
Reaction in the anterior chamber	0	2.5%
Late postoperative complications (after 14 days)		
Secondary cataract	6%	N/A
Transient increase in IOP	4%	0
Obstruction of istent/Preserflo	4.3%	0
Displacement of istent/Preserflo	2.6%	0
Blurred vision or disorders of vision	3.4%	0
Endophthalmitis	0	0
Filtering bleb scarring (at 6 and 12 months)	N/A	20%

Subconjunctival 5-fluorouracil injections were given to 8 patients from the Preserflo group (18.6%). The average dose of 5-FU was 5.0 ± 1.5 mg. In 6 patients from the Preserflo group (14%) the needling was performed. One patient from the Preserflo group was stitched up with additional suture, due to wound leakage. Two patients from the Preserflo group underwent reoperation due to filtering bleb fibrosis. In both cases, a classical trabeculectomy was performed.

No significant intraoperative complications were noted in the iStent group. In the immediate postoperative period microhyphema was observed in 8 eyes, all cases spontaneously resolved within a week of surgery without sequelae. Complications unrelated to iStent implantation occurred in 5 eyes. Four eyes developed posterior capsular opacification and were treated with Nd: YAG capsulotomy. One eye required intravitreal administration of an antiVEGF injections due to onset of exudative form of age-related macular degeneration. Immediately after the surgery, corneal oedema related to an increase in IOP was observed in 3 eyes on postoperative day 1, which resolved within 1 week. Viral corneal inflammation occurred in 1 eye at 6 months postoperative and was treated topically, resolving within 1 month with no visual complications. Four eyes in iStent group required additional glaucoma surgery.

## Discussion

The present study compared the efficacy and safety of MIGS and MIBS (implantations of the first-generation iStent combined with phacoemulsification vs Preserflo MicroShunt) in patients with POAG and PXG during a 12-month follow-up.

Both procedures effectively reduced IOP in POAG and PXG patients. iStent group achived better qualified success at 12 months for IOP criteria A < 18 mm Hg, wheather Preserflo group obtained better complete success at 12 months for IOP criteria B < 15 mm Hg. Surgical failure 12 months post op occured more fraquently in Preserflo group in comparison to iStent group (30% vs 6% respectively). There was a significant difference between groups in %IOP reduction after 12 months. Mean percentage reduction of IOP observed in Preserflo group was 33.5% vs 25.1% in iStent group, p = 0.028. The decrease in IOP was associated with a significant reduction in the postoperative use of antiglaucoma medications, down to 0.56 ± 1.08 and 0.46 ± 0.84 at 12 months in the Preserflo and iStent groups, respectively (p > 0.05).

While iStent implantation was not expected to produce the same IOP reduction as filtering surgery, our study revealed a comparable efficacy profile when considering main outcome measures. Additionally, more patients in the Preserflo group achieved an IOP of < 15 mmHg. This was expected because bleb-dependent procedures are known to produce lower IOPs. These findings are consistent with those found in literature, further demonstrating the capability of penetrating technique to achieve low target IOPs in a significant proportion of patients^16,17^. This is particularly important in patients with severe stages of disease.

Given the higher safety profile of iStent, coupled with a faster return to normal daily life, and comparable efficacy for moderate target IOPs (< 18 mm Hg), when combined with phacoemulsification, it presents a compelling case for its consideration. The number of clinically relevant complications was higher in the Preserflo group, with some adverse events requiring additional surgical intervention or conservative treatment. This positive benefit-to-risk assessment is particularly applicable for patients with mild-to-moderate glaucoma that does not yet warrant the risks of filtering surgery.

While the Preserflo MicroShunt and iStent were designed to normalize IOP through the drainage of aqueous humor from the anterior chamber, they differ in the implantation technique and mechanism of action. The iStent, implanted through the ab interno approach, facilitates the passage of aqueous humor through a natural outflow pathway. Considering the sources of increased resistance within the natural aqueous outflow pathway from the anterior chamber, one should consider not only the external wall of the Schlemm’s canal but also the openings of the collector channels that may play a significant role by changing the size of their entry depending on the IOP level. This hypothesis appears to be supported by the results of studies using more than one microbypass intraoperatively; in those studies, the decrease in IOP was not directly proportional to the number of stents inserted into the Schlemm’s canal. One possible solution could be to optimize the site of microbypass implantation, with the one with the largest number of collector channels being opened^16,18–22^. In the present study, the iStent was implanted into the nasal quadrant, which contained the highest average number of collector channels. The Preserflo MicroShunt uses the same aqueous outflow pathway as a trabeculectomy, using a filtering bleb to bypass the trabecular meshwork, considered the source of the highest resistance during aqueous outflow.^12^. The device is implanted using the ab externo approach, and the aqueous humor is drained through the new outflow tract to the suprascleral venous system or through the microcysts within the conjunctiva.

The intensive postsurgical care after Preserflo MicroShunt implantation deserves an attention. In a retrospective study conducted by Fea et al.^23^ analyzing the efficacy of MicroShunt in POAG and PXG patients, all complications were mild and resolved entirely after treatment. None of the complications posed a risk of vision loss. Postoperative subconjunctival injection of mitomycin C (MMC) or 5-fluorouracil (5-FU) without needling was required for 3 (2.9%) eyes. Needling was required in 19 (18.3%) eyes and revision surgery in 14 (13.5%) eyes. Four eyes (3.9%) required needling, followed by revision surgery. The mean time from surgery to first needling was approximately 30 days, whereas the mean time between surgery and surgical revision was 60 days. More promising results were obtained by Beckers et al.^24^, who observed transient early postoperative hypotony in 11.1% of cases POAG cases, but no persistent vision-threatening ocular adverse events. A few patients required needling (2.5%), subconjunctival injection of 5-FU (2.5%), or surgical revision (6.2%) to maintain the target IOP after Preserflo MicroShunt implantation Pillunat et al.^25^ compared Preserflo MicroShunt implantation with trabeculectomy, and found that the frequency of postsurgical interventions in the trabeculectomy group was significantly higher than in the Preserflo group, with a similar efficacy of both methods in reducing IOP. At six months post-procedure, mean IOP values in the Preserflo and trabeculectomy groups were 10.8 mmHg and 10.3 mmHg, respectively. The study groups did not differ significantly in terms of mean postoperative reduction in IOP, peak diurnal IOP, or median diurnal fluctuations.

Regarding the safety outcomes, iStent surgery is in favour in comparison to MicroShunt Preserflo. In a comparative study of iStent implantation combined with phacoemulsification versus phacoemulsification alone conducted by Kozera et al.^18^, no clinically relevant intraoperative complications were found in any patient subjected to the combined procedure. In the immediate postoperative period, the authors reported microhyphema in the anterior chamber in five eyes from the iStent group and subconjunctival hemorrhage in one eye; in all six cases, the complications resolved spontaneously within a week of the procedure without sequelae. Other studies on the patients with POAG and PXG subjected to combined surgery: phacoemulsification with first-generation iStent implantation^18,19^ revealed similar satisfactory outcomes. No intraoperative complications were observed. The most common early postoperative complications are microhyphema and Descemet’s membrane folds. An early postoperative increase in IOP occurred in seven (10%) patients; however, the IOP did not exceed 25 mmHg in any of the patients and dropped within a week post-procedure. Hypotony, endophthalmitis, and other serious complications were not observed. During the late postoperative period, two patients presented with secondary cataracts and were treated successfully with a YAG laser; three patients showed a decrease in BCVA due to previously diagnosed age-related macular degeneration (AMD), two experienced iStent rotation, and one required trabeculectomy due to a persistent postoperative increase in IOP up to 26 mmHg.

Another issue is the effect of phacoemulsification alone on the postoperative decrease in IOP, as all eyes in the iStent group in our study underwent a combined procedure. According to different studies, it is averaged at 1.4 mmHg, 1.55 mmHg, 1.88 mmHg, 2.9 mmHg, 3.1 mmHg, and 4.9–5.3 mmHg^26–30^. Depending on the type of glaucoma, the utmost reduction in IOP occurs in angle-closure glaucoma and PXG glaucoma. Several theories explaining this effect are taken into account. Firstly, after removal of the lens, the position of the ciliary body reccess changes, similar to the treatment with parasympathomimetics^20,31,32^. Other possible mechanism involves a decrease in aqueous humor production due to the contraction of the lens capsule after phacoemulsification^28^. In addition, improvement of aqueous humor outflow through the trabecular meshwork and Schlemm’s canal is also considerated^31^. This mechanism is especially considerated in the case of PXG, since the pseudoexfoliating material is washed out during the procedure from the anterior chamber, especially from the trabecular meshwork, which facilitates the outflow of aqueous humor through the Schlemm’s canal. This hypothesis may also explain the postoperative gradual increase in IOP in this type of glaucoma, which is proportional to the reproduction and accumulation of pseudoexfoliative deposits in the anterior segment of the eye. Another theory suggests an improvement of uveoscleral outflow^33^, caused by an increased release of prostaglandins during the procedure: PGE-1 increases the outflow of aqueous humor by the conventional route, while PGF-2 by the alternative route^34^. Analyzing the above-mentioned studies, it becomes evident that the reduction in IOP after surgery has a strong reverse correlation with the depth of the prior chamber before surgery, the width of the filtering angle, and the initial IOP level.

The present study was not free from potential limitations. This was a retrospective study with a relatively small sample size and a short follow-up period of 12 months. Moreover, the iStent and Preserflo groups differed in some preoperative characteristics, with patients in the former being older, presenting with lower baseline IOP, and receiving fewer antiglaucoma medications. Additionally Preserflo was standalone procedure whereas iStent was combined with phacoemulsification. Finally, the implantation procedure in either group was not preceded by a washout period. However, to our knowledge, this is the first study to compare these two devices, and there are no previous reports on the effectiveness of the Preserflo MicroShunt in Caucasian populations.

The landscape of glaucoma surgical armamentarium involves a spectrum of tools, each with unique attributes. However, tailoring the most suitable surgery for a specific patient is not without challenges. The growing popularity of MIGS and the abundance of available techniques warrant research on the subtle differences between various surgical methods. In this context, the article introduces some opinions into the discussion. In summary MIGS (iStent) and MIBS (Preserflo) effectively reduced IOP and the postoperative use of medications in glaucoma patients. However trabeculectomy remains the gold standard for glaucoma treatment, with the possibly to additional regulating IOP through early suture lysis, needling, or administration of antimetabolites. MIGS are less effective than trabeculectomy yet safer, and act synergistically with phacoemulsification, and do not affect the outcome of future conventional glaucoma surgeries. MIBS are equally effective as trabeculectomy or slightly inferior but produce a more predictable hypotensive effect with better morphology of the filtering bleb and less intensive postoperative care. However, the path toward implementing meticulous treatment reccomendations demands further prospective studies with longer follow-up periods. These studies will offer a valuable resource for future research endeavors, ultimately advancing our surgical management of glaucoma. Such studies could help optimize treatment approaches for patients with glaucoma of various severities.

## Methods

This study was based on a retrospective analysis of the medical histories of all consecutive patients who qualified for glaucoma surgeries, either iStent implantation combined with phacoemulsification or Preserflo MicroShunt implantation, at the Department of Ophthalmology, Medical University of Bialystok, and operated between January 2019 and January 2022.

The study was conducted in accordance with the principles of the Declaration of Helsinki. The implantation procedure was approved by the Bioethics Committee of the Medical University of Bialystok (APK.002.581.2021). Informed consent was waived by the Bioethics Committee of the Medical University of Bialystok due to the retrospective character of the study. All patient data were anonymized before statistical analysis of the results.

The qualification criterion for the procedure was the inability to achieve the target intraocular pressure (IOP)^16^ in patients with either mild or moderate POAG or PXG, despite the implementation of maximally tolerated pharmacotherapy (up to three antiglaucoma medications). In the case of iStent implantation, glaucoma patients who reached the target IOP also qualified as long as they required scheduled cataract surgery. The contraindications for surgical treatment included angle-closure glaucoma (ACG), inflammatory glaucoma, neovascular glaucoma, traumatic glaucoma, angle recession, filtration angle dysgenesis, and posterior adhesions. Additional exclusion criteria were a history of glaucoma surgery and a follow-up period of < 12 months. The implantation was not preceded by a washout period.

The analysis included patient demographics, such as sex and age, along with preoperative characteristics, such as glaucoma type, baseline IOP, best-corrected visual acuity in Snellen decimal (BCVA), number and type of antiglaucoma medications, mean deviation (MD), and pattern standard deviation (PSD) on the preoperative 30–2° Humphrey visual field (VF) test. Glaucoma severity was classified as mild (MD > -6 dB), moderate (MD − 6 to − 12 dB), or severe (MD < − 12 dB).

### Surgical technique

Implantation of the iStent was performed simultaneously with phacoemulsification, as described previously^35^, with the cataract procedure followed by implantation of the device to the trabecular meshwork within the inferonasal quadrant.

Implantation of the Preserflo MicroShunt was performed as a single procedure using the technique described in our previous study^13^.

All surgeries were performed under topical anesthesia with additional conjunctival administration of 2% Xylocaine in Preserflo MicroShunt group.

All patients received topical antibiotics postoperatively three times daily for seven days and topical steroids four times daily; the latter was gradually tapered over a 4–5-week period. Antiglaucoma medications were discontinued after the procedure, but when needed, they were prescribed again following EGS rules^16^.

The analysis included data from control visits before the procedure and at 6 and 12 months thereafter. The main outcome measures were: (1) success rates (defined as follows complete—no need for repeated glaucoma surgery, and IOP ≤ 18 mmHg (criterion A), ≤ 15 mmHg (criterion B) or 20% reduction from baseline^36^, and discontinuing all antiglaucoma medications; qualified—no need for repeated glaucoma surgery, IOP ≤ 18 mmHg (criterion A), IOP ≤ 15 mmHg (criterion B) or 20% reduction from baseline, with discontinuation of all antiglaucoma medications or without; (2) mean reduction (%) in IOP, (3) medication burden, (4) number of complications and additional interventions. Surgical failure was defined as the need for another glaucoma surgery at any time after the primary procedure or an IOP > 18 mmHg during two consecutive control visits (at 6 and 12 months) despite administering antiglaucoma medications.

### Statistical analysis

Sample size estimation: A minimum of 42 eyes were required to detect a 2.5 mmHg between-group difference in the mean decrease in IOP at twelve months after the procedure, with a significance level and 0.80 power of 0.05, assuming a standard deviation for the IOP decrease in both groups at 4.0 mmHg^37^.

Analysis was performed using the R package version 4.1.2. Categorical variables are summarized as numbers and percentages, and numerical variables as arithmetic means, standard deviations, medians, and interquartile ranges. The normal distribution of the study variables was verified using the Shapiro–Wilk test and confirmed based on skewness and kurtosis values. The homogeneity of variances was verified using Levene’s test. Between-group comparisons were based on Pearson’s chi-square test, Student’s t-test for independent groups, Welch’s t-test for independent groups, and the Mann–Whitney U-test. The significance of within-group changes over time was verified using t-tests for dependent groups and Wilcoxon’s test. Statistical significance for all analyses was set at α = 0.05.

## Data Availability

The datasets used and/or analysed during the current study available from the corresponding author on reasonable request.
